# A shortage of oral morphine in Egypt

**DOI:** 10.2471/BLT.15.156240

**Published:** 2016-01-01

**Authors:** Samy A Alsirafy, Dina E Farag

**Affiliations:** a Palliative Medicine Unit, NEMROCK, Kasr Al-Ainy School of Medicine, Cairo University, PO Box 99 Manial Al-Rodah, Cairo, 11553, Egypt.

Egypt, with more than 88 million residents in 2015 and an estimated 5-year cancer prevalence of more than 215 000 cases in 2012,^1^^,^^2^ has effectively no oral morphine. The lack of effective and affordable analgesia is catastrophic for people with end-stage cancer. Breast, liver and bladder cancers are the most common types of cancer in Egypt, and about half of cancer patients in the eastern Mediterranean Region only visit a physician when their cancer has reached an advanced – and often incurable – stage.^2^^,^^3^ For these patients, the focus of care is quality of life and the only realistic treatment option is palliative care.^4^

Medical treatment is the main element in cancer pain management and for most patients, relatively inexpensive drugs like morphine are effective.^5^ For many years, morphine has been on the *World Health Organization* (WHO) *model list of essential medicines* as the strong opioid of choice because of its suitability for management of moderate to severe cancer pain.^6^ However, global data on licit opioid consumption shows very low levels in many countries, suggesting that pain control may be inadequate for a large number of patients worldwide.^7^ The International Narcotics Control Board ranked Egypt 117 out of 178 countries for its level of consumption of narcotic drugs.^7^ From 2011–2013, the average consumption of narcotics was 75 defined daily doses for statistical purposes (S-DDD) per million inhabitants per day in Egypt.^7^ For comparison, the average consumption of narcotics in the top three ranked countries was 51 374 S-DDD per million inhabitants per day in the United States of America, 29 067 in Canada and 25 273 in Germany.^7^

For more than two decades, the only form of oral morphine registered in Egypt has been slow-release morphine tablets (30 mg), manufactured under licence by a single supplier in the United Kingdom of Great Britain and Northern Ireland, packed by an Egyptian company, distributed by an Egyptian governmental trading company then dispensed by pharmacies. For unknown reasons, in late 2014, oral morphine became unavailable in any form in Egypt. This does not appear to have been due to cost, since fentanyl, hydromorphone and oxycodone are still available. In the absence of oral morphine, these formulations are the only locally-available alternative to oral morphine, but they are more expensive and are therefore unaffordable for many cancer patients in Egypt.

Immediate action should be taken to make oral morphine available in Egypt. WHO advises governments to avoid such shortages through national policies that support cancer pain relief, educational programmes for the public, health-care personnel and regulators, and by modifying laws and regulations that limit the availability of opioid analgesics.^5^

We argue that morphine should be made available in Egypt in different forms and concentrations, consistent with WHO’s model list of essential medicines.^6^ To avoid stock-outs in the future, the country needs more than one source. Legislative changes are also required to remove the current restrictions limiting the amount of oral morphine in a single prescription to 420 mg. This is an inadequate supply for most patients with cancer-related pain.^8^^,^^9^ The Egyptian opioids control policy should be revised to achieve a balance between ensuring availability for medical use and preventing the misuse of these critically important drugs.^10^
